# Stem Cells and Gene Therapy in Progressive Hearing Loss: the State of the Art

**DOI:** 10.1007/s10162-020-00781-0

**Published:** 2021-01-28

**Authors:** Aida Nourbakhsh, Brett M. Colbert, Eric Nisenbaum, Aziz El-Amraoui, Derek M. Dykxhoorn, Karl Russell Koehler, Zheng-yi Chen, Xue Z. Liu

**Affiliations:** 1 Department of Otolaryngology–Head and Neck Surgery, University of Miami Miller School of Medicine, 1120 NW 14th Street, 5th Floor, Miami, FL 33136 USA; 2 John P. Hussman Institute for Human Genomics, University of Miami Miller School of Medicine, Miami, FL 33136 USA; 3 Medical Scientist Training Program, University of Miami Miller School of Medicine, Miami, FL 33136 USA; 4 Unit Progressive Sensory Disorders, Institut Pasteur, INSERM-UMRS1120, Sorbonne Université, 25 rue du Dr. Roux, 75015 Paris, France; 5 Department of Otolaryngology-Head and Neck Surgery, Boston Children’s Hospital, Harvard Medical School, Boston, MA 02115 USA; 6 Department of Otology and Laryngology, Harvard Medical School and Eaton-Peabody Laboratory, Massachusetts Eye and Ear Infirmary, Boston, MA 02114 USA

**Keywords:** Presbycusis, Ototoxicity, Autosomal dominant hearing loss, Gene therapy, Induced pluripotent stem cells

## Abstract

Progressive non-syndromic sensorineural hearing loss (PNSHL) is the most common cause of sensory impairment, affecting more than a third of individuals over the age of 65. PNSHL includes noise-induced hearing loss (NIHL) and inherited forms of deafness, among which is delayed-onset autosomal dominant hearing loss (AD PNSHL). PNSHL is a prime candidate for genetic therapies due to the fact that PNSHL has been studied extensively, and there is a potentially wide window between identification of the disorder and the onset of hearing loss. Several gene therapy strategies exist that show potential for targeting PNSHL, including viral and non-viral approaches, and gene editing versus gene-modulating approaches. To fully explore the potential of these therapy strategies, a faithful in vitro model of the human inner ear is needed. Such models may come from induced pluripotent stem cells (iPSCs). The development of new treatment modalities by combining iPSC modeling with novel and innovative gene therapy approaches will pave the way for future applications leading to improved quality of life for many affected individuals and their families.

## Introduction

### Hearing Loss

Hearing loss (HL) is the most frequent inherited sensory deficit in humans. The number of hearing-impaired individuals dramatically increases with aging. According to the World Health Organization’s estimate (http://www.who.int), the prevalence of hearing impairment will increase from approximately 460 million individuals in 2019 to over 900 million individuals by 2050. In particular, progressive, late-onset, non-syndromic, sensorineural HL (PNSHL), including age-related HL (ARHL, *aka* presbycusis), noise-induced hearing loss (NIHL), drug ototoxicity, and autosomal dominant (AD) HL, constitutes the most common causes of hearing impairment (Liu and Xu 1994; Liu et al. 1997; Yan and Liu, 2008b). PNSHL is a major health and socioeconomic burden on populations worldwide (Huddle et al. 2017). Presbycusis affects almost one out of every three individuals over the age of 65 years (Homans et al. 2017). In addition to a decrease in quality of life, the elderly population is particularly vulnerable to a decline in cognitive ability due to HL (Gates et al. 2010; Gurgel et al. 2014; Contrera et al. 2016; Su et al. 2017). Most forms of PNSHL occur due to the natural aging process; however, research has demonstrated that there are genomic factors that contribute or predispose an individual to PNSHL. Decades of research into the genetic etiologies of PNSHL have provided the necessary foundation to focus endeavors on developing targeted therapies.

### Cochlear Structure and Accessibility

The cochlea is a complex structure that enables the conversion of mechanical sound waves into electrical impulses that the brain can interpret. Sound enters the ear canal and first encounters the tympanic membrane (ear drum). Vibrations of this membrane then move the ossicles of the middle ear which in turn push on the oval window of the cochlea, generating a fluid wave in the spiral-shaped structure. This fluid wave causes the mechanosensitive hair cells of the cochlea to bend and initiate an action potential that is propagated to the brain stem.

**Fig. 1:**
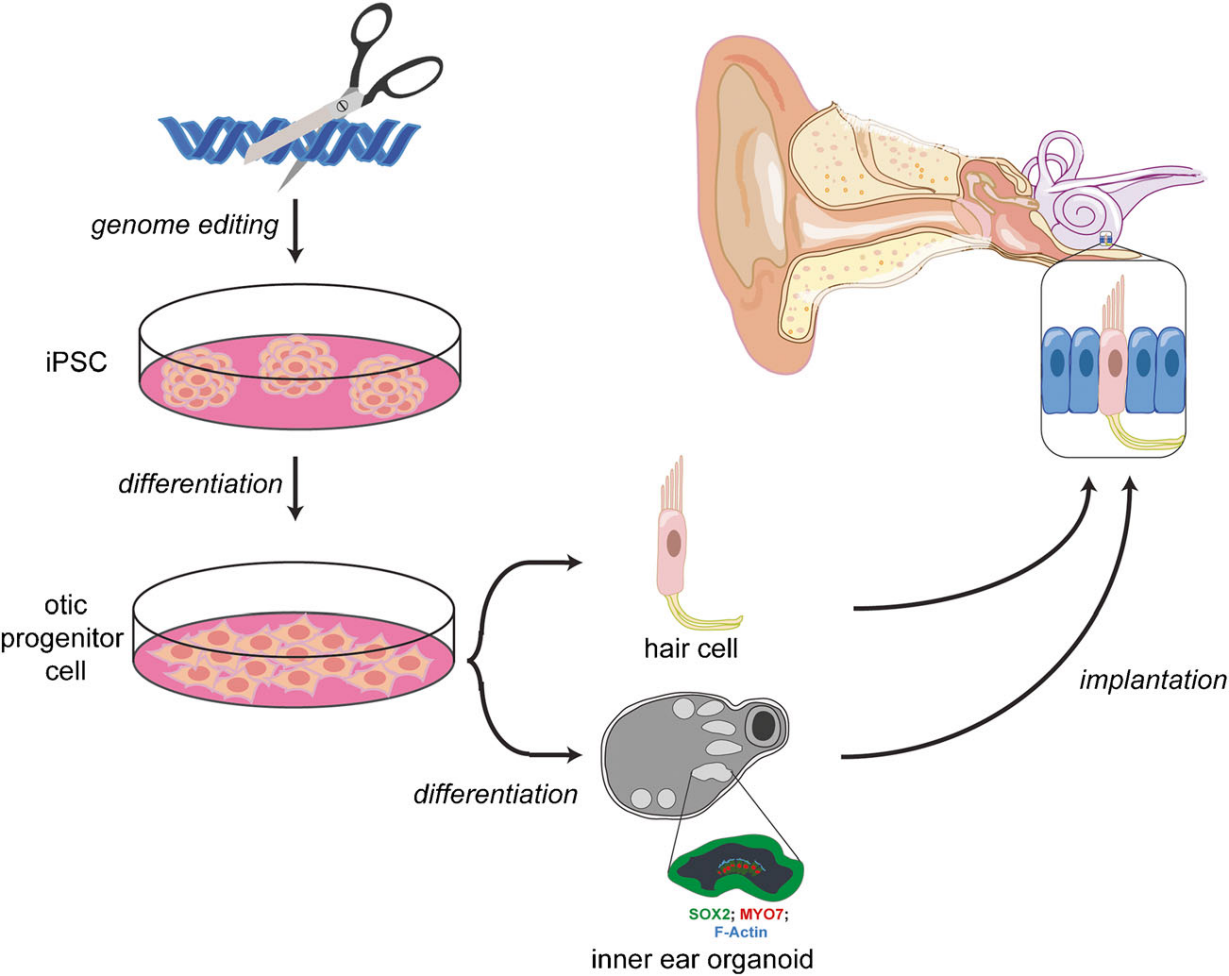
iPSC modeling and gene therapy in hearing loss. iPSCs are an effective tool for the modeling of genetic causes of hearing loss. They can be generated from individuals harboring a particular mutation, differentiated into 2D or 3D otic structures, and compared to controls. The cells also serve as a platform for experimenting with various gene therapy modalities on human tissue in vitro to guide further animal and clinical trials

The pathophysiology of PNSHL is directly related to hair cell death and dysfunction; however, there are no regenerative of preventative interventions for individuals known to have genetic or environmental predispositions. Gene therapy is emerging as promising treatment strategy for PNSHL. The ear is particularly amenable to local administration of therapeutic agents: there are surgical approaches to the cochlea including intratympanic injection, allowing diffusion across the round window membrane, round window membrane injection, stapedotomy with oval window injection, and cochleostomy or canalostomy with injection (Fig. 1) (Sacheli et al. 2013; Dai et al. 2017). A current clinical trial (NCT02132130) is using stapedotomy with oval window injection (Klickstein 2013; Dai et al. 2017).

A wealth of information on the inner ear pathophysiology of a large number of disease forms has been accrued, raising the possibility for targeted therapies. In this context, the delayed-onset and progressive nature of hearing impairment offers a suitable time window between genetic diagnosis and symptom manifestation, making PNSHL more amenable to preventative and protective therapeutic interventions. Development of gene- and cell-based therapies shows great potential to address PNSHL under a variety of conditions. Here, we focus on recent progress using induced pluripotent stem cells, iPSCs, to model inner ear-related deficits and the developments of gene therapy approaches to potentially treat the molecular mechanisms that underlie the loss of hearing.

## Therapies of the Future

### Stem Cells as Personalized Medicine

Stem cells are one promising avenue for developing a robust model of the human inner ear in vitro. Stem cells are defined by their capacity for self-renewal (i.e., they can continuously divide) and pluripotency (i.e., they can be differentiated into cell types from all three germ layers). The potential of differentiation in to any cell type makes stem cells attractive for therapeutic development and research approaches (Hans 2007).

#### Classes of Stem Cells

There are multiple types of stem cells defined by their origin and their potency. The first stem cells to be described and used in research settings were embryonic stem cells (ESCs). These are collected from the inner cell mass of the 5-day-old, pre-implantation blastocyst. ESCs have the potential to differentiate into any cell of the body and so have played an important role in understanding the nature of stem cells as well as in disease modeling and potential therapeutics. However, the widespread use of ESCs may not be feasible due to the limitations in harvesting, as well as ethical concerns over the destruction of embryos for research purposes (Daley et al. 2007; Sugarman 2008). Moreover, any therapy developed from ESCs would be non-autologous and, as such, face the risk of rejection if implanted into a patient (Wiley et al. 2015).

Another source of stem cells is adult stem cells (ASCs) which can be found throughout the body. Their function is to replace damaged cells and accelerate tissue healing (Davanger and Evensen 1971). While the lack of immune rejection makes ASCs attractive for clinical work and translational research, their limited differentiation capacity and ability to self-renew make them difficult to work with in the laboratory (Alison et al. 2000; Gage 2000; Li et al. 2003).

A very promising avenue of work is the innovative application of induced pluripotent stem cell (iPSC) technology. This new line of research was first described by Yamanaka in 2006 when his group was able to reprogram mouse fibroblasts into stem cells through the transgenic expression of a limited cocktail of transcription factors whose normal expression is restricted to stem and progenitor cells (Takahashi and Yamanaka 2006). This ground-breaking technique won the Nobel Prize in medicine in 2012. They were then able to replicate the work using adult human fibroblasts the following year (Takahashi et al. 2007). iPSCs have many of the same characteristics as ESCs making them as versatile as ESCs, but without the complication of rejection since they are generated from the patient’s own cells.

#### Induction of iPSCs

The induction of iPSCs is accomplished by transfecting the cells, obtained from skin punch biopsy, urine, or blood, with a limited set of transcription factors associated with stem and progenitor cell populations (Takahashi and Yamanaka 2006; Zhao et al. 2009). The most common cocktail of transcription factors used for reprogramming includes *Oct4*, *Sox2*, *Klf4*, and *c-Myc*. However, additional transcription factors (e.g., Lin28) and even miRNAs (e.g., miR-302) have been used to successfully reprogram somatic cells to iPSCs (Lin et al. 2008; Hanna et al. 2009). After 3 weeks of culturing, a high nucleus/cytoplasm ratio is noticeable, and iPSC markers (Oct4, Sox2, Nanog, Ssea4, Tra-1-60, Tra-1-81) are detectable by immunocytochemistry and quantitative real-time PCR (qRT-PCR). Karyotype analysis is necessary to verify that there are no chromosomal abnormalities. Viable mice can be formed from iPSCs by tetraploid complementation (Zhao et al. 2009). Embryoid body and teratoma formation (when injected into immunocompromised mice) are also examined as a sign of pluripotency. Further verification of pluripotency comes from observing markers for each of the three germ layers (e.g., Afp and Sox17 for the endoderm; Brachyury and Msx1 for the mesoderm; and Paz6 and Map2 for the ectoderm) (Chen et al. 2016).

One pitfall of this technique is that the retroviral vectors that were first used to induce iPSC generation can be randomly inserted into the host’s genome opening the door for insertional mutagenesis and oncogenic transformation (Okita et al. 2013). This problem has been circumvented by producing therapeutic grade iPSCs using either non-integrating viruses or virus-free induction methods. These methods introduce the reprograming molecules by way of mRNA, recombinant proteins, or miRNAs (Kim et al. 2009; Warren et al. 2010; Miyoshi et al. 2011). Another issue was that surgical skin biopsy is invasive. Later studies have shown that iPSCs can be successfully derived using somatic cells derived from blood or urine samples, which expanded their further clinical use (Staerk et al. 2010; Zhou et al. 2012).

### iPSCs in Hearing Research

iPSCs have been found to be useful in the study of hearing loss. Generating mice that have the same mutations as seen in a population can be expensive, time-consuming, and impossible in some cases. Patient-derived iPSCs are used in disease modeling to overcome this difficulty (Ye et al. 2009; Chen et al. 2016). Cellular modeling of deafness can be achieved for any given HL-associated pathogenic variant, regardless of the cellular or functional target, e.g., hair bundle morphogenesis, ion homeostasis, extracellular matrix composition, or transcription factors involved in cochlear development and hair cell generation (Yan and Liu, 2008a; Hilgert et al. 2009; Bademci et al. 2020).

#### 2D Cultures

Established and defined protocols have been developed to control mouse PSCs differentiation into inner ear cells, both under 2D and 3D conditions (Oshima et al. 2010; Koehler et al. 2013; Schaefer et al. 2018) (Table 1). Plating of the differentiating iPSC onto mitotically inactivated chicken utricle stromal cells resulted in the production of cell clusters containing mechanosensitive hair cell-like cells (Oshima et al. 2010). Using human embryonic stem cells, Chen et al. were able to develop differentiation protocols to obtain two types of otic progenitors: otic epithelial progenitors (OEP) that formed hair cell-like cells and otic neural progenitors (ONP) that formed auditory-like neurons (Chen et al. 2012).

**Table 1:** Studies that have generated protocols for in vitro, stem cell-derived, 2D, and 3D cultures

Reference	Cell type	Differentiation
Oshima et al. 2010	Human ESC/iPSC	2D hair cell-like cells
Chen et al. 2012	Human ESC	2D otic epithelial and neural progenitors
Koehler et al. 2013	Mouse ESC/iPSC	Organoid vestibular hair cells
Tang et al. 2016	Human iPSC	2D hair cell-like cells
Koehler et al. 2017	Human iPSC	Organoid vestibular hair cells
Schaeffer et al, 2018	Mouse ESC	Organoid vestibular hair cells
Jeong et al. 2018	Human iPSC	Organoid vestibular/cochlear-like hair cells
Chen et al. 2018	Human iPSC	2D otic progenitors for transplantation

More recently, Tang et al. used iPSCs from a deaf patient with a HL-associated variant in the *MYO7A* gene and corrected the variant using CRISPR/Cas9 technology (Tang et al. 2016). They were able to demonstrate that this genetic correction led to functional and morphological recovery of hair cell-like cells that were generated from patient iPSC lines. This study demonstrates the power of using genome-based editing in stem cells to develop an in vitro model for the study of the pathogenesis of sensorineural HL-associated genetic variants. Several more studies have used iPSCs to model a particular etiology of hearing loss such as the mouse *Barhl1* (Zhong et al. 2018; Hou et al. 2019), *pendrin* (Hosoya et al. 2019), *MYO7A* (Tang et al. 2016), and *MYO15A* (Chen et al. 2016).

#### 3D Cultures

The differentiation approaches employed by Chen et al. and Tang et al. produced hair cell-like cells that expressed some markers of native hair cells yet lacked the typical cell body and hair bundle morphology of native hair cells. More robust modeling of the human inner ear with stem cells was later accomplished using 3D organoid cultures. Koehler et al. generated organoids from human iPSCs by modulating *FGF*, *BMP*, *TGF*, and *WNT* signaling in cell aggregates suspended in Matrigel to generate 3D otic vesicle-like structures (Koehler et al. 2017). Over the course of 2 months, these structures developed into inner ear organoids with sensory regions containing hair cell-like cells targeted by neuronal projections. The development of iPSCs with 2A-GFP inserted at the stop codon of the *ATOH1* gene enabled the visualization of hair cells following organoid formation. Using this tool, the researchers demonstrated that organoid hair cells displayed electrophysiological properties similar to those seen with native hair cells. The organoids also had a winding, convoluted, and chambered appearance reminiscent of the inner ear labyrinth (Koehler et al. 2017). However, all of the published protocols to date appear to generate only vestibular, and not cochlear, hair cell-like cells.

Using a similar induction approach to Koehler et al., a recent study reported the generation of both vestibular and cochlear-like hair cells in a human organoid system (Jeong et al. 2018). The induction of cochlear-like hair cells was thought to be caused by upregulation of endogenous sonic hedgehog (Shh), known for its importance in cochlear hair cell development, as well as tighter control of FGF2 signaling early in the differentiation. The organoids in this study displayed the expected potassium channel activity. However, this activity did not fully recapitulate that seen in mature cochlear hair cells. Cochlear identity was determined using a limited set of gene and protein markers, and hair bundle morphology was not thoroughly examined. Some of the cells did not display tip links as would be expected from mature hair cells. The authors proposed that perhaps the cells had not reached full maturity and that steps such as using FGF20, 3, or 10, or retinoic acid in the differentiation protocol—or even acoustic stimulation—could assist in producing more mature hair cells in inner ear organoids (Jeong et al. 2018). Thus, it remains unclear how robust, cochlear-like hair cells can be generated in organoid systems. Recent work from Roccio and colleagues showed that cochlear organoids with hair cells could be generated from fetal tissue specimens; however, the routine use of these difficult to obtain samples for gene therapy testing may not be feasible (Roccio et al. 2018). While these studies have made significant contribution to developing a robust protocol for organoid generation, additional improvements are needed to further our understanding of these in vitro cultures and the production of mature auditory hair cells (Table 1).

### Gene Therapy for PNSHL

Gene therapy for hearing loss requires delivery of exogenous DNA or genome editing agents into the inner ear. These therapies are distinguished on whether they edit a target cell’s genome or supply nucleic acid sequences to express a protein that otherwise may be mutated. CRISPR/Cas9-based approaches have emerged as having promising therapeutic applications in humans (Delmaghani and El-Amraoui 2020).

#### CRISPR/Cas9 as the Future of Treating Genetic Disease

In 1987, several species of bacteria were found to display genomic regions with unknown function that contain regularly spaced, repeated sequences separated by variable regions (Ishino et al. 1987). Twenty years later, with improved sequencing technology and access to more extensive sequence databases, the variable regions between the repeats were identified as portions of bacteriophage genomes, and the involvement of these regions in bacterial immune system to counteract phages’ attacks was shown (Barrangou et al. 2007).

#### CRISPR Mechanics

CRISPR stands for clustered regularly interspaced short palindromic repeats. Researchers found that, within the genome of bacteria, there are CRISPR loci which contain repeated sequences of DNA (repeats) separated by sequences that correspond to viral DNA (spacers). The CRISPR-associated proteins (Cas) are endonucleases that can recognize viral DNA and are able to degrade the foreign invader (Jinek et al. 2012). A portion of this viral DNA is integrated into the genome of the cell at the CRISPR locus between the CRISPR repeats. When the DNA is transcribed, it forms crisprRNA (crRNA), which is composed both of the CRISPR repeat and the spacer corresponding to the integrated viral DNA. This two-part crRNA then binds to the Cas9 protein (Jinek et al. 2012). This protein-RNA complex is able to bind to viral DNA sequences corresponding to the highly specific RNA guide of the crRNA and carry out double-stranded cleavage of the viral DNA, thereby preventing it from harming the cell (Jinek et al. 2012). The precision of DNA cleavage, along with its facility of introduction, thus makes CRISPR/Cas9 an ideal tool for genetic engineering in eukaryotic cells. This has been first successfully used in human and mouse cells (Cong et al. 2013) and was soon followed by the successful production of knockout mice (Wang et al. 2013). More recently, CRISPR was used to cure Duchenne muscular dystrophy in mice upon post-natal retro-orbital viral-mediated injections. Despite the fact that CRISPR/Cas9 did not achieve full coverage of the mutated gene, the gene editing approach was sufficient to alleviate the disease symptoms (Long et al. 2016).

The *Staphylococcus pyogenes* Cas9 is the most frequently used Cas enzyme in mammalian gene editing. It can be programmed to introduce a double-stranded cleavage in DNA at a specific location by a roughly 100 bp, synthesizable, single guide RNA (sgRNA). The targeted region corresponds to the first 20 bp of the sgRNA. This region in the genome must be flanked by a protospacer adjacent motif (PAM). Each Cas enzyme requires a different PAM sequence, and this must be considered when designing a CRISPR protocol. Specifically, the PAM sequence for Cas9 is NGG (Jinek et al. 2012).

#### CRISPR Delivery

Once a target genomic region is identified and a guide generated, the gRNA/Cas enzyme hybrid has to be delivered into the cells. In vitro, electroporation is the most common delivery technique. While it is inefficient, CRISPR-corrected cells can be selected and clonally expanded (Hashimoto and Takemoto 2015). Microinjection is also used but is a time-consuming approach. The most popular and clinically promising delivery method is a viral vector delivery (Ablain et al. 2015; Long et al. 2016). Viruses that target the cell or tissue of interest can be engineered and packaged with the CRISPR machinery. An older method of delivery recently applied with great success to CRISPR is cationic lipid-mediated transfection (Felgner et al. 1987). Positively charged lipid spheroids interact and complex with negatively charged DNA or RNA. The lipid is then able to fuse with the membranes of cells and deliver their content.

#### DNA Repair in CRISPR Gene Editing

Once a CRISPR double-stranded break has been introduced at the target site, there are two methods of DNA repair. The cell can perform non-homologous end joining in which the break is sealed by annealing the loose ends of the DNA strand (Lieber 2010). This method is sufficient when disruption of a gene is desired. However, if one wants to change the function of a gene, or correct disease-associated variants, homology-directed repair is necessary. In this method of DNA repair, the cell is able to fix the break by incorporating a sequence from a highly homologous source template. In the case of CRISPR, donor DNA of about 100 bp can be introduced along with the CRISPR machinery. The donor DNA is completely homologous to the region surrounding the double-stranded break and flanking the new sequence that is desired to repair the mutated gene (Chu et al. 2015).

#### Possible Issues with CRISPR Gene Therapy

For all the benefits of CRISPR, a potential downside is the possibility of off-target effects. The technology has been improved to the point that these are increasingly uncommon and potential sites are predictable (Gurumurthy et al. 2016; Kleinstiver et al. 2016; Slaymaker et al. 2016). As a proof-of-concept, direct injection of CRISPR/Cas9 reagents into one-cell mouse embryos has been used successfully to correct a mutation in *Cdh23* that predisposes mice to age-related hearing loss with reversal of phenotype and without detection of any off-target effects by whole genome sequencing (Mianné et al. 2016).

#### Non-genome Editing Gene Therapy

Delivery of exogenous genetic material or therapeutic molecules like a neurotrophic factor are examples of non-genome editing therapeutic strategies (Sacheli et al. 2013). RNA interference (RNAi) can also be used to knock down gain-of-function mutations without altering the genome in any way (Mukherjea et al. 2010). This is accomplished by short interfering RNA (siRNA) or micro RNA (miRNA) which both bind complementary, target mRNAs, and induce mRNA degradation (Sharp 1999). Antisense oligonucleotides (ASOs) function similarly but are modified DNA bases that bind and regulate mRNA translation (Rossor et al. 2018). The transient nature of these treatment modalities makes them useful in instances where only a temporary effect is needed, such as concomitant treatment with ototoxic drugs or foreseeable noise exposure (Izumikawa et al. 2005; Mukherjea et al. 2010).

### Virally Mediated Therapies for PNSHL

A popular delivery method for gene therapeutics has been viruses such as adeno-associated viruses (AAV). AAVs, unlike lentiviruses, do not incorporate the foreign DNA into the host genome, mitigating the risk of gene disruption and off-target effects (Kesser et al. 2008; Sacheli et al. 2013; Chien et al. 2015). AAVs have been used successfully in sense organs such as the eye (Maguire et al. 2008; Testa et al. 2013; MacLaren et al. 2014; Edwards et al. 2016). For example, AAV vectors have been used to treat Leber congenital amaurosis safely showing improvements in vision that have been sustained throughout 3 years of follow-up (Maguire et al. 2008; Testa et al. 2013). In human trials of AAV gene therapy for choroideremia, one-third of patients showed immediate improvement that was stable at 3.5-year follow-up (MacLaren et al. 2014; Edwards et al. 2016).

Due to similarities between the cochlea and retina (isolated organs with immune privilege), increasing efforts have been focused on hearing loss treatment strategies using AAVs. Several studies using AAVs have shown success in delivery of gene products to the inner ear in animal models (Shu et al. 2016).

#### Gene-Specific, Viral-Mediated Therapies

Gene therapy using different types of AAVs has been successfully carried out in various hearing loss models. The AAV1 vector containing *Vglut3* was delivered to the cochlea of *Vglut3* knockout mice, which is a model of DFNA25. This delivery resulted in the restoration of ABR thresholds (Akil et al. 2012). Using another type of viral vector, AAV8, a single delivery of the *Ush1g* cDNA to the cochlea of newborn mutant mice reestablishes the expression and normal targeting of the Usher syndrome type 1G protein. This led to restoration of the architecture and mechanoelectrical sensitivity of hair cell bundles, thereby improving hearing thresholds, and durably and totally rescues the balance deficits of the treated defective mice (Emptoz et al. 2017).

A popular vector for delivery to the inner ear has been AAV Anc80L65. It was used in an Usher syndrome type 1C mouse model. The vector showed high transduction efficiency in outer hair cells and restored the balance and hearing defects of the *Ush1c* mice (Pan et al. 2017).

AAV-mediated delivery of *Tmc2* was able to rescue the hearing loss of *Tmc1* mutations in mice that are analogous to AR DFNB 7/11 and PNSHL DFNA 36 (Askew et al. 2015).

In a recent study, the Anc80L065 vector encoding *Tmc1* was also used to rescue hearing and vestibular phenotypes of DFNB7/11 recessive deafness mouse model (Nist-Lund et al. 2019).

Yeh et al. recently targeted *Tmc1* in the recessive Baringo mouse model harboring the *c.A545G* mutation. Due to the mutation type, they were able to use a modified Cas9 nickase enzyme that is linked to cytidine deaminase which allows for a single base pair modification to repair the *c.A545G* mutation without a double-stranded break. This cytosine base editor system was then packaged into a dual AAV system for delivery to the inner ear at post-natal day 1 (Yeh et al. 2020). The system showed a greatly increased efficiency of base editing (51%) compared to HDR (typically < 2 %) as well as restoration of hair cell morphology and function and increase in low frequency hearing up to 4 weeks (Yeh et al. 2020). Other genes that have been targeted by recent studies have been *whirlin* (György et al. 2019) and *clarin-1* (Geng et al. 2017; Dulon et al. 2018).

A synthetic AAV named AAV-inner ear (AAV-ie) has been shown to efficiently transduce cells of the inner ear. It was generated by inserting three, cell-penetrating peptides into the VP1 capsid of AAV-DJ. The newly generated AAV-ie was found to have a particular tropism for the supporting cells of the inner ear, transducing up to 77% of supporting cells in vivo when delivered by round window injection to mice. The authors then used AAV-ie to deliver the *Atoh-I* gene—known to cause supporting cells to transdifferentiate into hair cells—and found that it induced the growth of as many as 82 new hair cells per 100 μm (Tan et al. 2019).

RNAi has been used in the cochlea through viral delivery. In the DNFA36 gain-of-function mutation in *Tmc1*, fondly known as the *Beethoven* mouse, a single injection of a viral vector carrying an siRNA led to significant restoration of hearing (Shibata et al. 2016).

#### Gene-Independent, Viral-Mediated Therapies

Viral gene therapy has potential for common forms of PNSHL such as presbycusis, NIHL, and medication-induced ototoxicity where the therapeutic aim is regeneration of hair cells and not the correction of a single pre-existing genetic defect. For example, ATOH1 (also known as HATH1) is a transcription factor important in human hair cell differentiation and has shown therapeutic benefit in mammalian models of NIHL and ototoxicity (Izumikawa et al. 2005; Yang et al. 2012; Richardson and Atkinson 2015). Izumikawa et al. showed that *Atoh1* gene therapy with an AAV vector improved hearing in mature mice deafened by ototoxic medication exposure by inducing the transdifferentiation of non-sensory cells into hair cells (Izumikawa et al. 2005). Yang et al. showed significant improvement in ABR thresholds with round window injections of *Atoh1* with an AAV vector 1 week after noise exposure in a guinea pig model of NIHL (Yang et al. 2012). Some studies, however, have yielded conflicting results. Atkinson et al. used AAV vectors to deliver *Atoh1* alone or in combination with *Bdnf* or *neurotrophin-3* through a cochleostomy in guinea pigs deafened by a combination of loop diuretics and aminoglycosides (Atkinson et al. 2014). While they found that treated inner ear cells expressed markers of hair cell differentiation, there was no decrease in ABR thresholds at 3-week post-treatment (Atkinson et al. 2014). It is difficult to compare these studies as the disease models may represent different degrees of hair cell death; however, it can be speculated that *Atoh1* may be involved in survival of damaged hair cells rather than regeneration.

In a study in mature mice, Atoh1 was overexpressed in supporting cells at different developmental stages in transgenic mice (Liu et al. 2012). The authors were able to demonstrate that ectopic expression of Atoh1 led to hair cell differentiation in neonatal and juvenile mice in contrast to the adult mice where the supporting cells no longer responded to ectopic Atoh1 expression. In this study, the Atho1-HA^+^ mice were treated with kanamycin at P30 and started ectopic expression with tamoxifen at P33 and analyzed at P63. Although most supporting cells expressed Atoh1-HA, no new hair cell was evident, and the drug-induced hearing damage was not improved when analyzed by ABR at P63 and P83 (Liu et al. 2012). This pre-clinical data demonstrates the limitation of Atoh1 gene therapy. There is a loss in the ability of Atoh1 to convert supporting cells to hair cells in mice post-natally which likely parallels early gestational stages in humans.

In the ongoing phase one and two clinical trial, NCT02132130, the use of *ATOH1* gene therapy in adults with severe to profound bilateral PNSHL is being investigated. In the study, the adenovirus vector Ad5 is being used due to the fact that it has no size restrictions and no risk for insertional mutagenesis (Chien et al. 2015). As discussed previously, short-term expression of ATOH1 has been sufficient to induce transdifferentiation of the supporting cells into hair cells in pre-clinical animal studies. The goal of this clinical trial is to evaluate the safety of the Ad5 injections and to study and the efficiency of supporting cell transdifferentiation (Staecker et al. 2011).

Another clinical trial NCT03996824 is also exploring AAV transduction of hair cells collected from patients undergoing non-conservative surgery for vestibular schwannomas. The study aims to show the efficacy and safety of viral transduction in vitro. Successful transduction will be assessed by immunofluorescence, and if shown to be safe, these studies (clinicaltrials.gov) may pave the way for other viral gene therapy trials.

### Non-virally Mediated Therapies for PNSHL

Non-viral delivery techniques may be desirable when short-term effect is needed, as in the case of ototoxic drug exposures. This can be accomplished by a variety of methods.

#### Antisense Oligonucleotides

In addition to miRNA or siRNA for RNAi, antisense oligonucleotides (ASOs, 20-30 bp sequences complementary to the target mRNA) can be used to achieve the knockdown of deleterious gene products or in the case of dominant mutations. ASOs backbone structure is modified to evade degradation by DNAses and allow sustained therapeutic effects. After binding to the target mRNA, they can induce degradation of the transcript by endonuclease digestion, prevent translation by inhibiting ribosome recruitment, or prevent pre-mRNA splicing (Holmlund 2003). Lentz et al. were able to successfully use an ASO to interfere with the hearing loss and vestibular deficit caused by aberrant splicing in *Ush1c*, encoding for the Usher 1C harmonin protein (Lentz et al. 2013). Upon ASO injections, the authors could show an increase in expression of the normal protein, a better stereocilia organization, hair cell rescue, and improved vestibular function in the treated *Ush1c* mice. The beneficial effects were stable for several months, showing that ASO therapy is a viable option to target genetic causes of hearing loss (Lentz et al. 2013). In this study, the ASO was delivered by systemic injection, which is less than ideal for use in humans. However, ASOs can also be delivered to the cochlea locally as has been previously described for other therapeutic approaches.

#### Cochlear Electroporation

Cochlear electroporation refers to the delivery of genetic material into inner ear cells through the use of a current that temporarily increases the permeability of cell membranes. Pinyon et al. demonstrated that a cochlear implant could be harnessed to use for electroporation and transduce the implanted cochlea of guinea pigs deafened by ototoxic agents with a naked DNA construct coding for *Bdnf* (Pinyon et al. 2014). This approach boosted the growth of spiral ganglion neurites in the direction of the cochlear implant electrodes and improved cochlear implant performance as measured by ABR recordings (Pinyon et al. 2014). More recent studies have demonstrated that direct delivery of BDNF and neurotrophin to the round window after a prolonged 95 dB sound stimulus leads to improved preservation of ABR thresholds (Sly et al. 2016). The use of gene therapy for the continuous endogenous production of neurotrophic factors thus may be protective in environmental and genetic causes of progressive hearing loss with a broad range of etiologies and may improve prosthesis performance when combined with a cochlear implant.

#### Lipid-Mediated Delivery

Liposomes are an alternative packaging system to deliver gene products to the inner ear. They protect the nucleic acids from cleavage by nucleases and increase permeability across the plasma and nuclear membranes (Dass 2002).

One study that showed success using lipocomplexes to target cells of the inner ear introduced siRNA targeting a mutant allele of *Gjb2* in mice. This approach achieved 70% decreased expression of the mutant *Gjb2* without any effect on endogenous, wild-type *Gjb2* (Maeda et al. 2005).

In a pre-clinical study, Gao et al. used cationic lipocomplexes to deliver the CRISPR/Cas9 system programmed to target *Tmc1* in the *Beethoven* mouse (Gao et al. 2018). The recovery of ABR thresholds was not sustained throughout development. Continued inner ear hair cell degeneration was observed. A subsequent study delivered the SaCas9-KKH using an AAV vector that was able to improve the ABR thresholds in *Beethoven* mice for up to a year (György et al. 2019).

### Cell Transplantation

As discussed, a three-dimensional organoid model is an excellent tool to model the pathophysiology of genes involved in hearing loss and to develop novel therapeutic approaches. However, it is also important to develop strategies to use the iPSC-derived progenitor cells in recovery of hearing loss phenotype in vivo. Transplantation of otic neural progenitors in an in vivo auditory neuropathy animal model led to significant improvement in auditory brain response (ABR) thresholds (Chen et al. 2012).

In a recent study, iPSC-derived otic epithelial progenitor cells, expressing GFP, were transplanted into the Slc26a4-null mice. After 4 weeks of transplantation, GFP expression was found in MYO7A-positive cells at the site where inner hair cells were absent. In addition, fibers of spiral ganglion neurons that were in the vicinity of hair cell-like cells expressed synaptophysin strongly suggesting synaptic formation with transplant-derived hair cells (Chen et al. 2018). This study suggests the ability of using iPSC-derived progenitor cells in vivo and provides the possibility of utilizing corrected patient iPSC-derived progenitor cells in clinical therapy although it does not address the possibility that the engrafted cells transferred the GFP to the native cells (Table 1).

## Conclusions and Future Directions

PNSHL is a prime target for the development of gene therapeutics. Current and future studies will pave the way for human trials to determine the efficacy of gene delivery and genome editing in treating hearing loss. The focus of gene therapy research recently has been on utilization of patient-derived iPSCs as a model and therapeutic tool in the treatment of genetically caused hearing loss. In addition, the efficiency of CRISPR/Cas9 genome editing in correction of mutant alleles has led to major advances in treatment of hearing loss in multiple animal models. The current state of research has demonstrated that the combination of iPSC modeling and CRISPR/Cas9 genome editing shows great potential of stem cell gene therapy in the cochlea (Fig. 1). There is hope that we will see a viable therapy in trials for genetic HL derived from these techniques in the coming years.
